# Reviewing experimental studies on chemical thermal energy storage in Cementitious composites: report of the RILEM TC 299-TES

**DOI:** 10.1617/s11527-025-02803-w

**Published:** 2025-10-13

**Authors:** Lorena Skevi, Xinyuan Ke, Stéphane Ginestet, Claudiane Ouellet-Plamondon, Fernando Gomes, Martin Cyr

**Affiliations:** 1 Department of Architecture and Civil Engineering, University of Bath, Bath, UK; 2 Laboratoire Matériaux et Durabilité des Constructions (LMDC), Université de Toulouse, INSA, UPS, Toulouse, France; 3 Department of Construction Engineering, University of Quebec, École de Technologie Supérieure, Montréal, Canada; 4 Instituto de Macromoléculas Professora Eloisa Mano (IMA), Federal University of Rio de Janeiro, Rio de Janeiro, Brazil

**Keywords:** Thermochemical energy storage, Ettringite, Geopolymer, Experimental test

## Abstract

Thermochemical energy storage (TCES) is a method of storing energy by using reversible chemical reactions to absorb and release heat. TCES materials generally possess the highest volumetric energy density and negligible heat losses during cyclic charging/discharging when compared with sensible and latent heat storage materials. The controllable charging/discharging processes in the TCES materials make them suitable for long-term or seasonal thermal energy storage, which can help improve the resilience of the existing energy system and built environment. In recent years, there has been a growing number of studies on the use of cementitious materials as low-cost and low-carbon thermochemical energy storage materials, including ettringite, calcium aluminate cements, and geopolymers. In this study, the state-of-the-art development using cementitious materials for thermo-chemical energy/heat storage applications is reviewed and systematically compared in terms of their compositions, energy storage operating conditions, and energy storage performance. Technical recommendations are proposed for standardised characterisation and testing protocols of these cementitious (composite) materials used for thermochemical heat storage. The current research challenges and future research needs in this field are also discussed.

## Introduction

Solar energy can provide more than three times the total primary energy supplied globally, as estimated by the International Energy Agency (IEA) [1]. Under clear-sky conditions at solar noon, the intensity of solar radiation on the Earth's surface can reach approximately 1000 W/m^2^ [1]. However, this value fluctuates significantly depending on weather conditions, geographic location, and time of year. Moreover, it is difficult to match the peak energy consumption hours (mostly after sunset) related to human activities with the peak solar energy hours, or to cover energy needs during wintertime when sunlight is limited. Therefore, there is a need for energy storage systems that can effectively utilise this abundant solar energy by storing it for interseasonal energy supply. Long-term energy storage is also needed for lower-grade thermal energy sources such as the waste heat that occurs from various industrial processes and which cannot always be utilised on-site [2, 3].

Thermochemical energy storage (TCES) describes the process by which thermal energy is stored in a material and can be released from it at a later time through reversible physical–chemical reactions [4]. Thus, two main cycles are distinguished in the process; the charging cycle, during which the material is heated and thermal energy is stored in the bonds of the material through an endothermic reaction of decomposition, and the discharging cycle during which the reversible exothermic reaction is favoured under certain conditions and the stored energy is released. The process is summarised in (1 [5], where AB represents a solid reactant that decomposes to phases A and B upon heating (charging cycle); A is the solid phase in which the heat is stored, B is the released phase, most commonly a gas, and ${n}_{A}, {n}_{B}, {n}_{AB}$ are the stoichiometric coefficients). In the reverse reaction, heat is released when A and B come in contact (discharging cycle).

1 $${n}_{AB}\bullet AB\left(s\right)+heat \leftrightarrow {n}_{A}\bullet A\left(s\right)+ {n}_{B}\bullet B(g)$$

The energy remains stored in the material until the conditions for the activation of the exothermic reactions that will initiate the discharging process are met. This process is characterised by sorption phenomena during which a gas (sorbate) is absorbed into the bulk or adsorbed on the surface of a solid or liquid material (sorbent) [3]. The molecules of the sorbate gas are bound to the sorbent by physical intermolecular forces (mainly Van der Waals and hydrogen bonding) and/or by stronger chemical bonds, the formation of which results in the heat release [6].

**Fig. 1:**
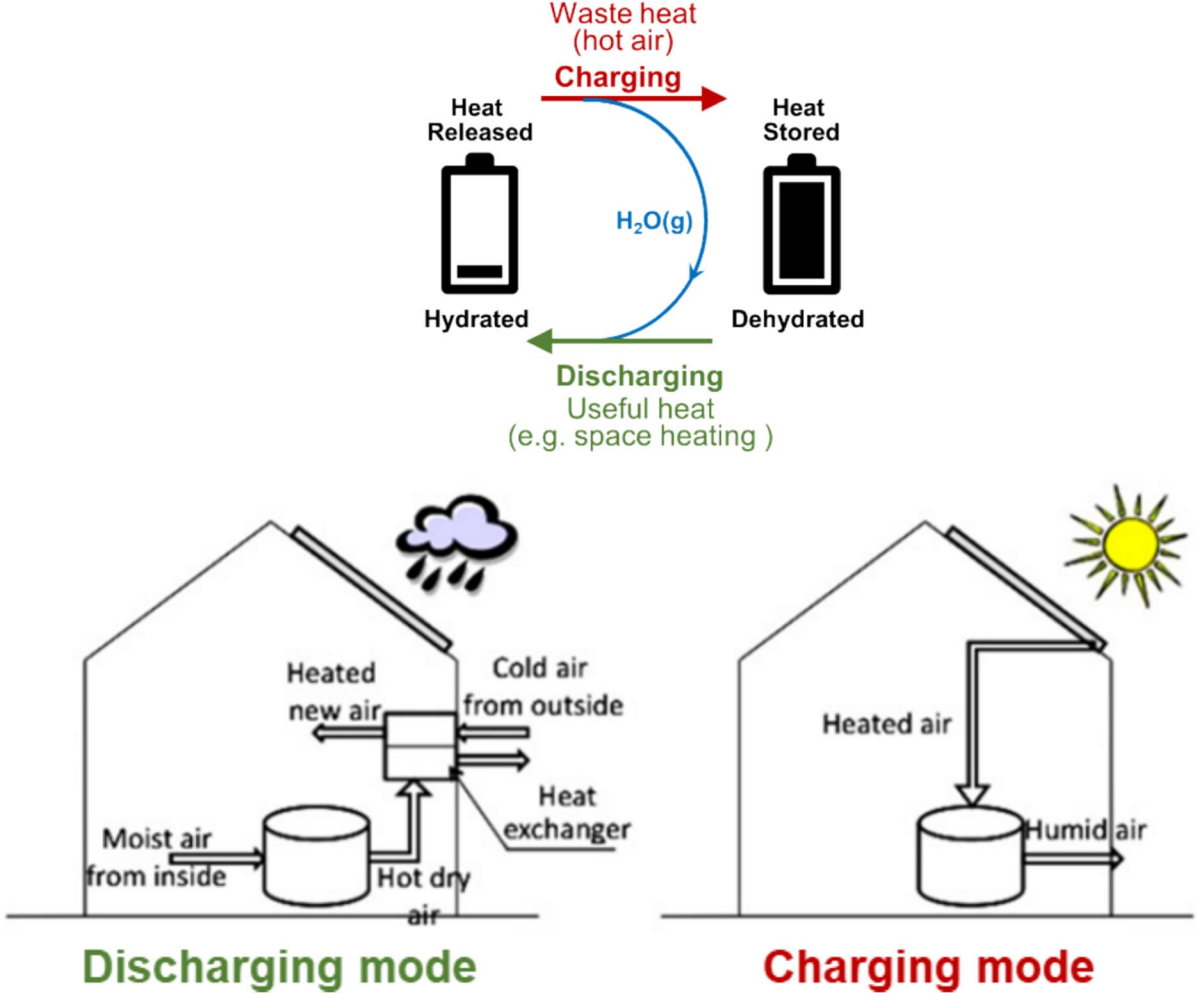
**A** Working principles of water sorption TCES (top) and heat storage (top). **B** Domestic heat storage: heat release (bottom) [9]

Water vapour is the most common gas used in thermochemical sorption reactions, especially for domestic interseasonal thermal energy storage, as it permits operation at low charging and discharging temperatures [7, 8]. Thus, for water sorption/desorption reactions, the AB in Eq. 1 normally represents hydrated salts, minerals, zeolites, and other thermochemical energy storage materials, while A indicates their dehydrated form and B the released water vapour. Figure 1A illustrates the working principle of water sorption TCES, where with the supply of heat, i.e. waste heat in hot dry air form, the thermochemical energy storage materials release water and store the heat within them as chemical energy; with the supply of water, i.e. cold moist air, the dehydrated thermochemical energy storage materials react with the water and discharge part of the stored heat as the reaction proceeds. The application of this mechanism in domestic heat storage/release is shown in Fig. 1B.

**Fig. 2:**
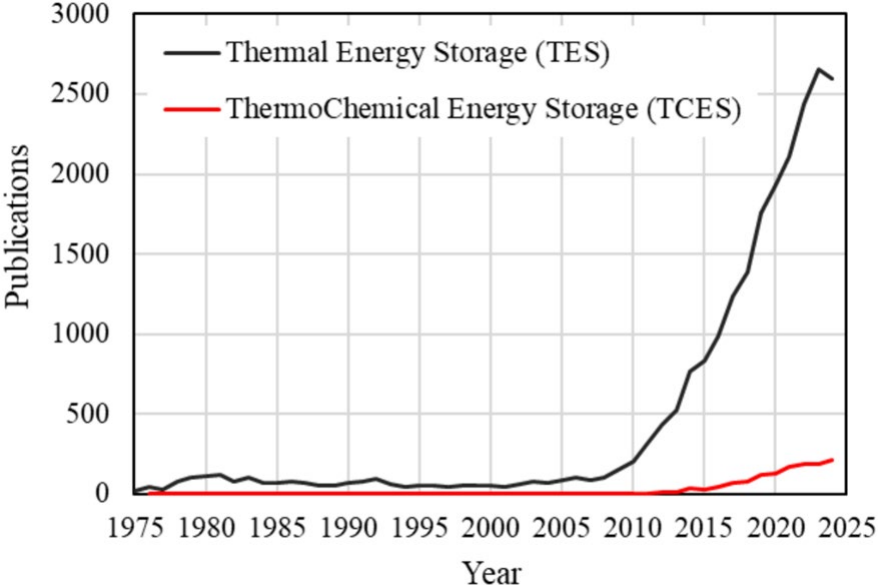
Publication progress on the research of thermochemical energy storage (TCES) compared to the overall research on thermal energy storage (TES). Source: Scopus, search “thermal energy storage, and “thermochemical energy storage” for TES and TCES curves respectively. Database last accessed on 11/11/2024

Apart from long-term thermal energy storage, TCES also entails higher energy density per volume of material and negligible heat losses compared to sensible and latent heat storage, which suggests that a lower volume of materials can be used to achieve similar energy storage capacity [10–12]. The controllable charging/discharging processes in TCES materials make them suitable for long-term/seasonal thermal energy storage, improving the resilience and efficiency of the existing gas-centred and the emerging fully electric energy in buildings [13]. Although the thermochemical capacity of certain materials has been known since the 1960s [3], a growing interest in this research field has primarily emerged over the past decade due to the pressing need for decarbonisation of the energy and heat sector. This is reflected in the increasing number of publications on TCES since 2010, as shown in Fig. 2. It also demonstrates that this heat storage pathway is newer and less explored considering the overall research in thermal energy storage, which mainly concerns the more traditional methods of sensible and latent heat storage.

Often in the literature, the term sorption energy storage is used to describe the energy storage processes during which physical sorption rather than chemical phenomena prevail [14], while in other cases, the term is used interchangeably with the term thermochemical energy storage. In this review, the term thermochemical energy storage is used to describe both the sorption and the chemical storage processes. In addition, different classifications of the TCES have been proposed in the literature over the past decade, the variations of which led Solé, Martorell [15] to the conclusion that further studies are needed to establish a clearer classification. The prevailing tendency is to categorise TCES based on the reactions that enable the thermal energy storage and release. In this direction, [16] distinguished two broad categories of TCES, the first of which involves sorption phenomena, including adsorption and absorption, whereas the second involves chemical reactions. However, as noted by [17], adsorption concerns both physical and chemical bonding, known as physisorption and chemisorption respectively, therefore, this categorisation might not draw an accurate distinction between the different TCES systems.

A categorisation of TCES based on the type of sorbent material has also been proposed [14, 18]. According to this, solid sorbents such as silica gel, zeolites, activated carbon and natural rocks are mainly involved in adsorption phenomena during the charging/discharging cycles, therefore, the energy in this case is stored and released through gas–solid reactions. On the other hand, in the case of liquid sorbent/sorbate pairs, such as the LiBr/H_2_O, KOH/H_2_O, and CaCl_2_/H_2_O absorption is the prevailing mechanism and hence gas–liquid reactions take place. Salt hydrates/water vapour and ammoniates/ammonia pairs make up the third category of chemical sorbents according to [18] and [14], Although these sorbent/sorbate pairs are also governed by solid/gas reactions, in this case, the chemical reactions that also take place outweigh the physical sorption processes, and due to the higher reaction enthalpies of the former higher heat storage density is achieved [14]. Finally, the fourth category concerns composite sorbents, which consist of solid sorbent materials (silica gel, zeolites etc.) loaded with chemical sorbents (i.e., salt hydrates). These composites combine the mechanical and chemical stability of the solid and the high storage capacity of the chemical sorbents resulting in stable TCES materials with high energy storage density [19]. Scapino et al. [20] further extended the previous classification, by distinguishing the chemical sorption reactions in weak chemisorption occurring when salt hydrates or ammoniates are the sorbents, and in strong chemisorption including metal hydride, redox and oxide-hydroxide or oxide-carbonate reactions [20]. The gas–solid phenomena taking place in the latter are characterized by the breakage and formation of stronger chemical bonds, entailing higher energy storage density compared to the decomposition (hydration/dehydration) reactions that occur in the salt hydrate and ammoniate sorbents.

**Fig. 3:**
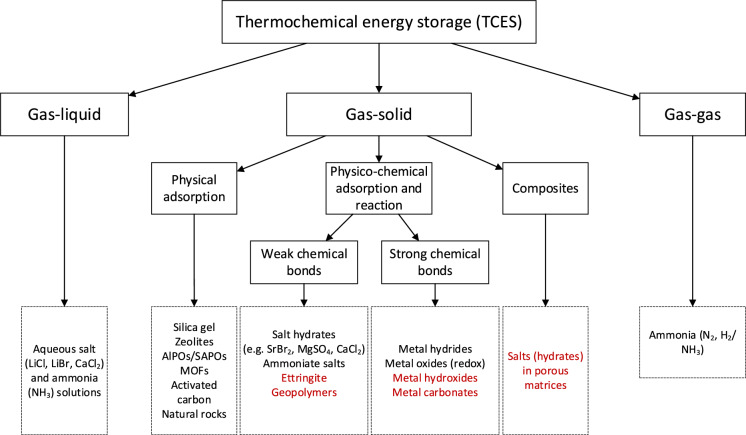
Proposed classification of the TCES processes and materials. The materials highlighted in red are (related to) cementitious materials that are of interest for this review and are discussed in more detail

Based on the above, a classification of the existing TCES systems is proposed in Fig. 3, which is developed by combining different sources from the literature [3, 14, 19–22], as each of them focused on one category or one part of the presented processes. The main materials of each category that have been used to date are also given as examples in Fig. 3.

Cementitious materials have been well-explored and applied in sensible and latent heat storage applications due to their low cost and durability at relatively high temperatures. In recent years, their use in TCES has emerged as a response to the need for low-cost, low-embodied carbon and easy-to-operate TCES materials [3, 23]. Such technical requirements are not fully met by more common TCES materials such as salt hydrates [24, 25], zeolites [26], carbonates [27], and hydroxides [28], despite their high energy density. Ettringite minerals [29–31], alkali-activated geopolymer materials [32, 33], and cement-based composites containing salt hydrates [2, 34] are the main examples of cementitious materials that have been investigated for TCES applications. Water vapour is commonly the gas phase in the gas–solid reactions that take place during the low-temperature charging/discharging of these materials. At the same time, limestone [10, 35], a widely used supplementary cementitious material (SCM), has been known for its TCES capacity through carbonation/decarbonation reactions, which take place at high temperatures (600 °C) with CO_2_ as the main gas involved.

Different functional thermochemical heat storage materials and physicochemical mechanisms are involved in these novel cementitious energy storage materials, resulting in diverse energy storage performance and optimal application conditions. This review paper provides a summary of the technical background of the thermochemical energy storage technology, commonly used materials, and prototype designs. The state-of-the-art research development using cementitious materials for thermochemical energy/heat storage applications is reviewed and systematically compared in terms of their compositions, working conditions, energy storage performance, and durability/longevity. Technical recommendations are proposed for standardised characterisation and testing protocols of cementitious (composite) materials used for thermochemical heat storage. The current research challenges and future research needs in this field are also discussed.

## Cementitious materials for thermochemical heat storage

In this section, the design of cementitious materials for TCES, including their operating conditions and heat storage performance, as well as the design of prototypes for different types of cementitious materials is systematically reviewed and compared. The sample preparation, materials characterisation, and energy performance evaluation methods applied to the thermochemical cementitious materials are also reviewed and discussed in this section. The cementitious materials include Portland cement-based materials, cementitious minerals, geopolymers, as well as source materials that exhibit hydraulic or pozzolanic behaviour, such as fly ash, blast furnace slag, and hydraulic lime. Table 1 summarises the performance of cementitious composites designed for TCES.

**Table 1:** Compositions, operating conditions and thermochemical energy/heat storage performance

Reaction type	Functional component	Solid reactant composition	Gas reactant	Experimental scale	Operating conditions (Temperature & RH range)	Thermal conductivity (W/m∙K)	Volumetric energy density (kWh/m^3^)	Enthalpy ΔH (kJ/moleH_2_O)	Heat capacity (J/kg∙K)	Reference
Dehydration/hydration	Ettringite	Various ettringite salts (Fe-, CO_3_-, Cl, NO_3_)	H_2_O vapour	Material	Charging: > 70 °C, RH 0% Discharging: 40 °C, RH 95%	n. d	n. d	37.8 (dehydration)	1000–1300	Struble and Brown [36]
		95% CSA cement + 1% Al powder + 4% Ca(OH)_2_ (w/c = 1.4)	H_2_O vapour	Material and reactor	Charging: 60 °C for 3 days, RH 0% (heated water passing through metal tube) Discharging: 20 °C, up to RH 100% for 3 days, (humidified nitrogen)	0.084	170 (theoretical) 138 (charging) 61 (discharging)	71.1 (hydration)	1260	Ndiaye, Cyr [29] Ndiaye, Ginestet [37]
		95% CSA cement + 1% Al powder and 4% Ca(OH)_2_ (w/c = 1.4)	H_2_O vapour	Reactor	Charging: 60 °C, RH 0% for 3 days (hot, dry nitrogen) Discharging: 20 °C, RH 100% for 3 days (humidified nitrogen)	0.084	170 (theoretical) 165 (charging) 117 (discharging)	71.1 (hydration)	1260	Ndiaye, Ginestet [30] Ndiaye, Cyr [38]
		CSA/gypsum (w/b = 0.60)	H_2_O vapour	Reactor	Charging: 110 °C, RH 0% Discharging: 20 °C, 0.55 m^3^/h of air	0.13	325 (charging) 61 (discharging)**	n. d	n. d	Kaufmann and Winnefeld [39]
		OPC + Calcium aluminate cement with calcium sulfate (w/b = 1.1)	H_2_O vapour	Reactor	Charging: 80 °C, 0% RH Discharging: 20 °C, RH 90%, flow rate: 3–5 m^3^/h N_2_	n. d	176 (discharging)	54.6	n. d	Chen, Johannes [40]
		Pure ettringite (synthetic, Ca(OH)_2_ + Al_2_(SO_4_)_3_∙14H_2_O)	H_2_O vapour	Reactor	Charging: 80 °C, 0% RH Discharging: 20 °C, RH 90%, flow rate: 0.003 m^3^/h N_2_	n. d	n. d	n. d	n. d	Chen, Johannes [41]
	Geopolymers	Metakaolin activated with NaOH + NaSiO_3_	H_2_O vapour	Material	Charging: 200 °C	n. d	350	83.8	1000–1400	Ke and Baki [32]
	Composites with salt hydrates	Portland cement with 21% MgSO_4_, w/c = 1	H_2_O vapour	Material	Charging: 140 °C	n. d	50 (discharging)	n. d	n. d	Lavagna, Burlon [34]
		Portland cement with SrCl_2_⋅6H_2_O (50 wt. %)	H_2_O vapour	Reactor	Charging: 150 °C for 1 h, airflow of 0.00025 m^3^/s Discharging: 18–20 °C, 60–70% RH	n. d	112	n. d	n. d	Clark and Farid [42] Clark and Farid [43]
		Metakaolin geopolymer with CaCl_2_	H_2_O vapour	Material	Charging: 200 °C	n. d	n. d	60–68	n. d	Skevi, Ke [33]
	Hydroxides	CaO	H_2_O vapour	Material	Charging: 400 °C-600°C	n. d	n. d	n. d	n. d	Funayama, Takasu [44]
		CaO	H_2_O vapour	Reactor	Charging: 400 °C-600°C	n. d	132–215	n. d	n. d	Schmidt and Linder [45]
		MgO	H_2_O vapour	Reactor	Charging: 310 °C-370°C Discharging: 140 °C-170°C	0.06	144	n. d	1600	Yan, Yang [46]
Decarbonation/ carbonation	Carbonates	CaCO_3_	CO_2_	Material	Charging: > 880 °C	n. d	1247	165.8	n. d	Humphries, Møller [47]
		MgCO_3_	CO_2_	Material	Charging: ~ 450 °C	n. d	943	96.7	n. d	Humphries, Møller [47]
		CaMg(CO_3_)_2_	CO_2_	Material	Charging: ~ 590 °C	n. d	540	125.8	n. d	Humphries, Møller [47]
		CaCO_3_	CO_2_	Reactor (small—only 2 g of sorbent)	Charging: 930 °C 50% of CO_2_ atmosphere	n. d	257	n. d	n. d	Teixeira, Afonso [35]
		CaCO_3_	CO_2_	Material	Charging: 750 °C in N_2_ atmosphere (5 min) Discharging: 850 °C in CO_2_ atmosphere (5 min)	n. d	838.9	n. d	n. d	Moreno, Arcenegui-Troya [48]

ǂ n. d. = not determined

*Calcium sulfoaluminate cement (containing 55% ye'elimite (4CaO·3Al_2_O_3_·SO_3_), 22% anhydrite (CaSO_4_)) – Ndiaye 2017, 2018: only the reactor set-up was improved to achieve higher heat storage performance (the material was kept the same)

**Mentioned in Chen 2021a

### Ettringite-based materials

In ettringite-based materials, the heat is stored when ettringite is dried at a temperature below 100 °C. As a result of the drying process, water is lost leading to the transformation of ettringite to meta-ettringite, a material with much lower water content (endothermic process), as shown in Eq. 2. When combined again with water, physical adsorption of capillary water takes place at the start of the hydration (Van der Waals bonds), and meta-ettringite rehydrates to form ettringite (Eq. 3). Both these physical and chemical processes lead to the release of heat (exothermic processes) [30]. At temperatures higher than 100 °C, ettringite is commonly converted to monosulphate and sulphate hemihydrate, or hydrogarnet [39], thus the dehydration stage should take place at the temperatures below this threshold.

2 $$\begin{gathered} {\mathbf{Charging}}: \hfill \\ {\text{Ettringite}}\left( {{3}0{\text{H}}_{{2}} {\text{O}}} \right) + {\text{Heat}} \to {\text{Metaettringite}}\left( {{\text{12 H}}_{{2}} {\text{O}}} \right) + {\text{Water}}\left( {{\text{18 H}}_{{2}} {\text{O}}} \right) \hfill \\ \end{gathered}$$

3 $$\begin{gathered} {\mathbf{Discharging}}: \hfill \\ {\text{Metaettringite}}\left( {{\text{12 H}}_{{2}} {\text{O}}} \right) + {\text{Water}}\left( {{\text{18H}}_{{2}} {\text{O}}} \right) \to {\text{Ettringite}}\left( {{3}0{\text{H}}_{{2}} {\text{O}}} \right) + {\text{Heat}} \hfill \\ \end{gathered}$$

As seen from Table 1, ettringite-based materials have the lowest operating temperatures among the cementitious materials (50–60 °C) [30, 39, 49], which makes them a particularly cost-effective heat storage system. However, since ettringite can be decomposed at temperatures above 100 °C, its application in environments where higher temperatures might occur (e.g., utilisation of waste heat from industrial processes) is limited. The volumetric energy density of the ettringite-based materials, referring to the heat released during the discharging cycle, varies between 61 and 176 kWh/m^3^ [29, 30, 37–40].

The susceptibility of ettringite-based heat storage systems to decomposition and carbonation under humid conditions and in the presence of CO_2_ has been emphasized by many studies as the main disadvantage of these materials [39, 50]. One possible approach to improve the durability performance is via compositional optimisation. Blending calcium aluminate cement with OPC (around 20wt.%) has shown improved resistance to carbonation under humid CO_2_ conditions, thereby preserving storage capacity over multiple cycles [50]. Compared to common energy storage materials like zeolites that have very fast sorption kinetics and achieve instantaneous sorption equilibrium within microseconds, due to their low thermal conductivity and thermal diffusivity [30], the sorption and desorption kinetics of ettringite-based materials are slow, which becomes more significant at large-scale applications where large quantities of the material are used [39].

Finally, there is a hysteresis (difference between the enthalpy of desorption and sorption) noticed in all studies where ettringite was used. Honorio et al. [31] studied the hysteresis of meta-ettringite sorption and found that the newly formed H-bonds contribute to the cohesion of the dried material, which made it difficult for water molecules to penetrate the structure upon rewetting, limiting its cyclic performance. However, using aerated or foamed calcium sulfoaluminate (CSA) cement can enhance water vapour diffusion and thermal transport, improving hydration/dehydration response times [51]. Since the development of ettringite-based thermochemical energy storage materials is still at an early stage, innovative approaches, such as incorporating thermally conductive additives and combining them with fast-response water sorbents, could further enhance their practical viability.

### Cement composites with salts

Salt hydrates are largely studied due to their high energy density. However, they are prone to deliquescence and lose mechanical stability, leading to low cyclability [34]. Also, it has been shown that salt powders can easily agglomerate during hydration and therefore a host matrix is needed to minimise agglomeration and swelling [25, 42]. Overall, when it comes to salt hydrates for thermochemical energy storage, chemical and mechanical stability seem to be the main technical challenges that need to be investigated [42].

The key features for the host material are porosity, mechanical stability, thermal conductivity, and economic viability [42]. The porous host matrix can be active or inactive in the thermal storage processes. Solid microporous sorbents, such as zeolites or silica gels, are characterised by a high level of hydrothermal stability, with higher power outputs and cyclability, but lower energy density and higher cost [34]. Cementitious materials are generally porous enough to host a considerable amount of salt but their porosity is not high enough to avoid problems with the water vapour flow during the charging cycle [34]. An improvement in the moisture diffusion can be achieved through the inclusion of thermally conductive inert materials such as expanded natural graphite (ENG) [25], which can increase the thermal conductivity of the composite cementitious material at the same time. It has been shown that thermal conductivity is positively correlated with the reaction rate. Therefore, the inclusion of ENG would enable faster release and transfer of the stored heat during the discharging process.

The salt-containing cement paste exhibits fast setting behaviour [34]. The salt-cement storage systems (Eq. 4) operate at low temperatures. The charging temperature changes according to the salt used: for example for SrCl_2_ it is 128 °C [42] and for MgSO_4_∙7H_2_O it is 80–140 °C [34]. Richter, Habermann [52] analysed the performance of 308 salts with a hydration temperature above 150 °C, and considered CaSO_4_ and SrBr_2_ the most promising with SrBr_2_ performing the best in terms of cyclability [25]. There are also salt hydrate based waste materials, like carnallite and bischofite, which are promising as they both contain the well investigated salt hydrate MgCl_2_•6H_2_O, so they could offer a promising alternative to the current concern regarding cost when using pure salt hydrates [25]. Almost all the studies devoted to low-temperature chemical energy storage (i.e., building applications) use salt hydrates [3, 6].

4 $${\text{Salt}} \cdot {\text{xH}}_{{2}} {\text{O}}\left( {\text{s}} \right) + {\text{Heat}} \rightleftarrows {\text{Salt}}\left( {\text{s}} \right) + {\text{xH}}_{{2}} {\text{O}}\left( {\text{g}} \right)$$

It is worth noting that, although the structural integration of salts into porous cementitious matrices may appear similar to strategies used in shape-stabilised phase change materials [53], the underlying mechanisms differ significantly. In the systems discussed here, energy is stored and released through reversible chemical reactions, mostly via salt hydration/dehydration, rather than through latent heat storage via phase changes.

### Geopolymers

The use of geopolymer composite materials for thermochemical heat storage is an emerging new field that has recently attracted attention from academics. The main component of geopolymer materials, alkali aluminosilicate hydrate (N-A-S–H gels) has the capacity to undergo cyclic dehydration-rehydration processes at a temperature below 200 °C, enabling the release and storage of heat as chemical potential [32]. During these processes, an energy storage capacity of 350 kW∙h/m^3^ (218 Wh/kg) at the charging temperature of 120 °C can be achieved [32]. The atomic-level investigation of the alkali-activated metakaolin using the neutron pair distribution function (nPDF) analysis also revealed that dehydration below 400 °C does not cause structural change to the aluminosilicate framework [54], suggesting that geopolymer materials might also be suitable for medium-to-high temperature thermal energy storage.

The thermochemical energy storage performance of geopolymers is largely governed by their chemical composition and porous structures. Alkali aluminosilicate hydrate gels with a lower Si/Al ratio result in higher maximal water uptake capacities (at equilibrium under RH 95%), while using sodium-based activators achieves a higher water uptake capacity when compared with activators with mixed alkalis (i.e., a mixture of sodium and potassium based activators) [32]. The use of siliceous activators could result in higher overall moisture diffusion coefficients, but may also lead to significant dehydration hysteresis between 20 and 40% RH conditions due to complex pore connectivity [32]. However, the delayed dehydration performance could be overcome by optimising the charging (dehydration) conditions and choosing relative humidity conditions below 20%. Balancing between the maximal water uptake capacity and the hydration/dehydration kinetics, sodium-based geopolymer gels with a bulk Si/Al ratio of around 1.5 exhibited promising performance as a standalone thermochemical energy storage material.

5 $${\text{Na}}_{{2}} {\text{O}} \cdot {\text{xAl}}_{{2}} {\text{O}}_{{3}} \cdot {\text{ySiO}}_{{2}} \cdot {\text{nH}}_{{2}} {\text{O}}\left( {\text{s}} \right) + {\text{Heat}} \rightleftarrows {\text{Na}}_{{2}} {\text{O}} \cdot {\text{xAl}}_{{2}} {\text{O}}_{{3}} \cdot {\text{ySiO}}_{{2}} \left( {\text{s}} \right) + {\text{nH}}_{{2}} {\text{O}}\left( {\text{g}} \right)$$

Thermochemical salt (i.e., CaCl_2_, MgSO_4_, K_2_CO_3_) impregnation might also have the capacity to further improve the thermochemical energy storage capacity of geopolymer composites [33], similar to the salt impregnated zeolite [55], expanded clay [56], and metal–organic framework (MOF) [57]. Better chemical and thermal stability during the energy storage processes has also been observed in these composite materials. When comparing the energy storage capacity and embodied carbon of commonly used materials for thermochemical energy storage, the plain geopolymers can achieve heat storage capacity comparable to zeolite-13X and MOFs, but only possess 10% and 7% of the embodied carbon (compared to zeolite-13X and MOFs). In comparison with some of the commonly used thermochemical salt hydrates for domestic heat storage (i.e., MgSO_4_, CaCl_2_ and K_2_CO_3_), the geopolymers exhibited similar storage capacity to CaCl_2_ and higher capacity than K_2_CO_3_, but lower embodied carbon per unit mass. The very-low embodied carbon and satisfactory heat storage capacity of the geopolymer materials make them extremely promising for high-performance, low-cost, thermally stable, carbon–neutral novel TCES composite materials. However, the fundamental understanding of the effects of intrinsic physical and chemical properties of the geopolymer materials on controlling their thermochemical heat storage performance is yet to be fully understood.

### Carbonates

The reversible reaction of calcium looping (CaL) has attracted more attention due to its high heat storage density (theoretically up to 3180 kJ⋅kg^−1^), high working temperature (650–1000 °C), non-toxic and low cost of heat storage materials such as limestone and dolomite [47, 58]. Due to the high operating temperatures, these calcination/carbonation reactions have been primarily considered for applications in concentrating solar power (CSP) plants in which solar energy up to 1000 °C is directed by the heliostat mirrors to the receivers [59].

During the decomposition of CaCO_3_ particles (calcination), CaO and CO_2_ are produced and stored separately. It should be noted that part of the endothermic energy that occurs during the decomposition translates into sensible heat energy in both reaction products, which can be instantly utilised with heat exchangers [60, 61]. The thermochemical energy is recovered by the exothermic carbonation reaction that occurs when bringing the stored CaO into contact with CO_2_, as described by Eq. 6.

6 $${\text{CaO}}\left( {\text{s}} \right) + {\text{CO}}_{{2}} \left( {\text{g}} \right) \rightleftarrows {\text{CaCO}}_{{3}} \left( {\text{s}} \right)\Delta {\text{H}} = - {\text{178 kJ}}/{\text{mol}}$$

The temperature at which the calcination/carbonation reactions take place, and their duration vary in the literature. According to Ortiz, Tejada [61], calcination temperatures above 930 °C are necessary for initiating decarbonation reactions in short residence times. However, harsh calcination conditions, with high temperatures and/or prolonged times may result in the sintering and agglomeration of the produced CaO particles, which would significantly reduce their surface area and as a result their reaction potential with CO_2_ during carbonation [60]. The pore clogging effect that takes place during carbonation and results from the deposition of the CaCO_3_ layer on the CaO particles is another key parameter to consider in the calcination/carbonation reactions, as the diffusivity of the CO_2_ in the CaO particles for their full carbonation is strongly dependent on this layer [62]. Therefore, mild conditions (relatively low temperature) are suggested during calcination, leading to more porous CaO particles, whereas fast carbonation kinetics are required for the maximum conversion of CaO to CaCO_3_ to take place before the reaction is controlled by the CO_2_ diffusion [60]. Similar observations were made by Setoodeh Jahromy, Jordan [10], where lowering the decomposition temperature from 1150 °C to 880 °C resulted in improved fly ash-CO_2_ reactions. While the calcination reaction of CaCO_3_ proceeds at above 900 °C in pure CO_2_ it can take place at 700–750 °C in pure He or a mixed CO_2_/He gas atmosphere [58]. The integration of He in the calciner enhances the thermal conductivity of the gas mixture and enhances the diffusivity of the produced CO_2_ in the mixture gas [60].

Benitez-Guerrero, Sarrion [60] found that the particle size of the natural carbonates can affect the pore structure of the CaO particles formed during calcination, and consequently the plugging of the pores with CaCO_3_ during carbonation. In CaCO_3_ systems (limestone and marble) with particle sizes larger than 45 μm larger pores were formed and were prone to clogging, while pores < 50 nm were formed in particle sizes below 45 μm, since sintering was reduced, allowing the diffusion of CO_2_. The study also found that the presence of the inert MgO in the natural carbonate materials, such as in dolomite, hindered the sintering and aggregation of the CaO particles, allowing for better CO_2_ diffusion even in larger particles [60]. On the other hand, particle sizes lower than 45–40 μm could result in cohesive powders with reduced flowability, which is important at the reactor scale [59]. Thus, the operating parameters must be optimized to reach a higher particle conversion and to avoid CaO-CO_2_ recombination into CaCO_3_ at the reactor outlet.

Apart from the presence of MgO inert domains, an arrest of sintering and agglomeration was also noticed in the presence of silicate impurities in CaO and MgO particles derived from natural dolomite, resulting in better cyclability of the reagents [47]. The same principle is followed with the addition of Al_2_O_3_ in CaO composites [63], while the injection of steam during calcination and carbonation reactions has also been shown to alleviate the sintering of CaO particles [64]. Recently, the possibility of direct solar absorption of Ca-based materials for their calcination has been explored, switching the focus on the solar radiation absorbance capacity of the materials [35, 48]. For further improvement of this property the doping of the carbonate materials with dark inert additives, such as sludge and SiC [35], MnFe_2_O_4_ [65], and carbide slag [58] has been investigated in conjunction with their counter-sintering effect.

### Hydroxides

The hydration/dehydration of metal oxide and hydrate pairs such as CaO/Ca(OH)_2_, (7), and MgO/Mg(OH)_2_, (8), is a TCES pathway that demands lower temperatures than the previously examined calcination/carbonation process, as charging temperatures (dehydration) are around 300–400 °C and discharging (hydration) close to 100–170 °C [46].

7 $${\text{CaO}}\left( {\text{s}} \right) + {\text{H}}_{{2}} {\text{O}}\left( {\text{g}} \right) \rightleftharpoons {\text{Ca}}\left( {{\text{OH}}} \right)_{{2}} \left( {\text{s}} \right) + \Delta {\text{H}} = - {1}0{4}.{\text{4 kJ}}/{\text{mol}}$$

8 $${\text{MgO}}\left( {\text{s}} \right) + {\text{H}}_{{2}} {\text{O}}\left( {\text{g}} \right) \rightleftharpoons {\text{Mg}}\left( {{\text{OH}}} \right)_{{2}} \left( {\text{s}} \right) + \Delta {\text{H}} = - {81}.0{2}\;{\text{kJ}}/{\text{mol}}$$

Schmidt and Linder [45] presented the energy balance in the oxide/hydroxide system during the charging and discharging processes, showed that approximately one-quarter of the heat released during CaO dehydration is sensible heat, with the remainder being thermochemical energy suitable for long-term storage. The low particle size of the raw CaCO_3_, approximately 5 μm, can cause problems regarding the flowability of the particles at the reactor scale and various approaches have been proposed for improving the reactor conditions [66]. To address the flowability and handling challenges of hydroxide-based materials in practical systems, recent research has explored pelletisation and granulation techniques to improve mechanical strength and reduce dust formation [11]. Further adaptation and optimisation of reactor designs could also help minimise the risk of agglomeration or segregation during thermal cycling [15]. However, a detailed discussion of reactor engineering is beyond the scope of this review.

## Test methods and conditions

Characterisation of the thermochemical storage materials can be examined at three different scales, at materials-level (small quantities of few milligrams), at reactor-level (larger quantities of few kilograms) and at system-level (full-scale projects) [16]. In this review, characterisation and evaluation methods for TCES materials from materials and reactor levels are reviewed and summarised.

### Material level

The experimental testing methods and testing parameters for characterising thermochemical energy storage materials are summarised in Table 2.

**Table 2:** Experimental testing methods and testing parameters for characterising thermochemical energy storage materials (all in powders)

Examined properties	Characterisation methods	Testing parameters	References
Thermal analysis	TGA	Charging: from room temperature to the calcination temperature (725 °C), at 300 °C/min under helium atmosphere Discharging: carbonation at 850 °C (temperature increase at 300 °C/min) under pure CO_2_ atmosphere for 5 min	Benitez-Guerrero, Sarrion [60]
		Charging: calcination at 750 °C under pure N_2_ for 5 min Discharging: rise of temperature up to 883 °C and carbonation under pure CO_2_ at atmospheric pressure	Ortiz, Valverde [27]
		Charging (calcination): 850 °C under a pure N_2_ atmosphere for 10 min (1 L/min) Discharging (carbonation): 850 °C under a pure CO_2_ atmosphere for 10 min (1 L/min)	Yang, Li [58]
	DSC-TGA	50–410 °C under N_2_ atmosphere (40 L/min) at 10 K/min	Ogorodova, Gritsenko [67]
		25–80 °C for 5 h, 10 K/min under N_2_ flow (50 mL/min)	Chen, Horgnies [50]
		DSC analysis, two heating ramps 25 °C for 30 min, then ramped from 25 °C to 395 °C at 10 K/min, held at 395 °C for 15 min and then cooled down to 25 °C, then held at 25 °C for another 15 min and ramped to 395 °C again at the same heating rate (second ramp)	Ke and Baki [32]
Water sorption	DVS	RT up to 400 °C and relative pressures from 0 up to 0.95	Lavagna, Burlon [34]
		Samples pre-dried at 200 °C, then RH 0%–95% at 25 °C	Ke and Baki [32]
		Multiple cyclic swing between 0%RH and 95%RH, for evaluating stability	Skevi, Ke [33]

#### Material composition

At material level characterisation techniques such as XRD, FTIR and Raman spectroscopy are commonly applied for determining the composition of the raw and the synthesised materials used for TCES. These methods can allow the monitoring of the potential changes in the material composition, providing insights into the degree of decomposition or recomposition of the materials during the charging and discharging phases.

#### Microstructure and pore characteristics

In addition to the composition, the microstructure of the materials, including their surface and pore structure properties, is crucial in the study of sorption phenomena. SEM images provide a view of the microstructure of materials, offering information on material density, pore structure, and connectivity, both of which can affect the kinetics of the gas sorption in the material and consequently the kinetics of the energy storage and release. More advanced imaging techniques such as CT-scanning can also provide information about the inner structure of the material without destroying it, as well as changes caused by repetitive cycles.

**Fig. 4:**
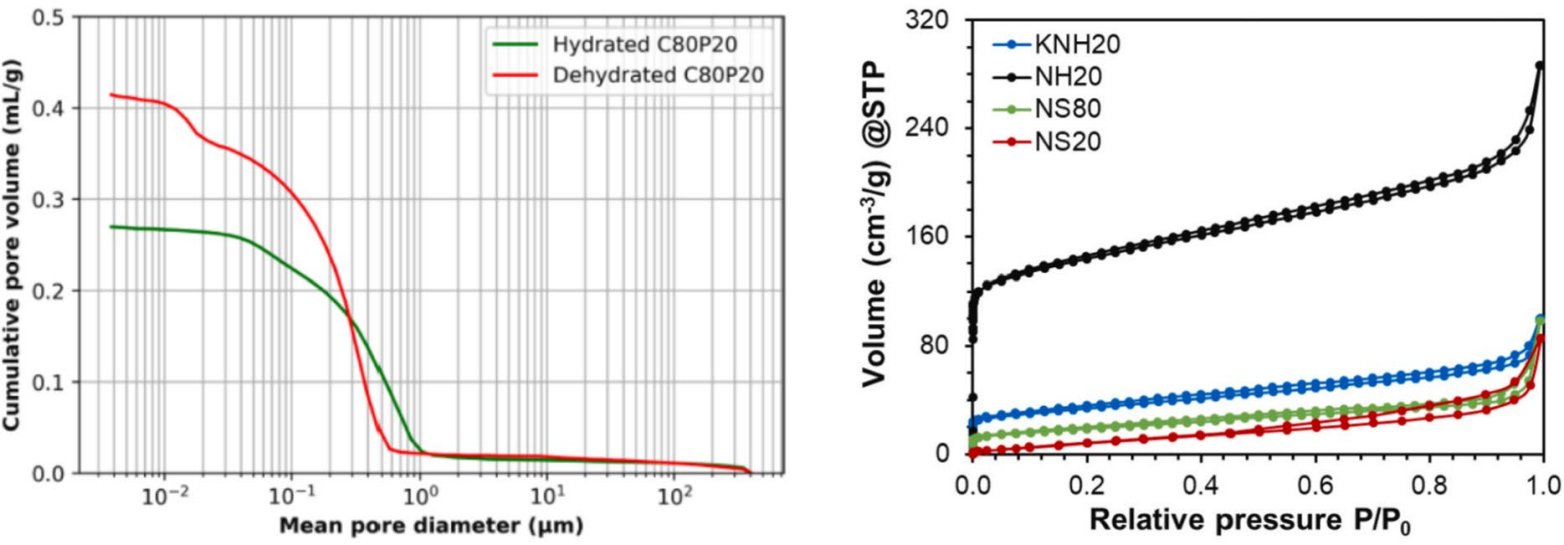
(left) pore volume of hydrated and dehydrated ettringite-based thermochemical energy storage materials measured by MIP [40]; (right) pore volume of different geopolymer-based thermochemical energy storage materials [32]

The pore structure of the bulk material is also studied and quantified with mercury intrusion porosimetry (MIP), which provides information on the size of the pores and their respective volume present in the material. It is considered that higher porosity will result in improved vapour sorption performance, and consequently higher energy efficiency of the TCES [40, 68]. However, the presence of voids in the material is also expected to lead to the reduction of thermal transfer properties such as thermal conductivity, thus leading to lower charging/discharging rates [69]. In addition to MIP, which focuses on the mesoscale pores range (Fig. 4 left), N_2_ sorption provides information for the porosity of the material at the nanoscale (Fig. 4 right). This includes calculating the specific surface area using the Brunauer–Emmett–Teller (BET) multipoint method [47] and determining pore size distribution and volume in the macropore (> 50 nm) and mesopore (2–50 nm) ranges [47]. In [35], the average absorptivity (%) was also calculated.

The high temperatures of the CaL process affect the textural properties of the Ca-rich materials, and there is an expected relation between the CaO carbonation conversion and SBET and pore volume, i.e., the lower values of SBET and pore volume were observed for the materials with lower CaO carbonation conversion [35]. In addition, repeated carbonation cycles can result in textural (surface) changes of the material (a reduction in SBET observed after 10 cycles) meaning that pore blocking may be responsible for the decrease of CaO carbonation [35].

#### Water vapour sorption

**Fig. 5:**
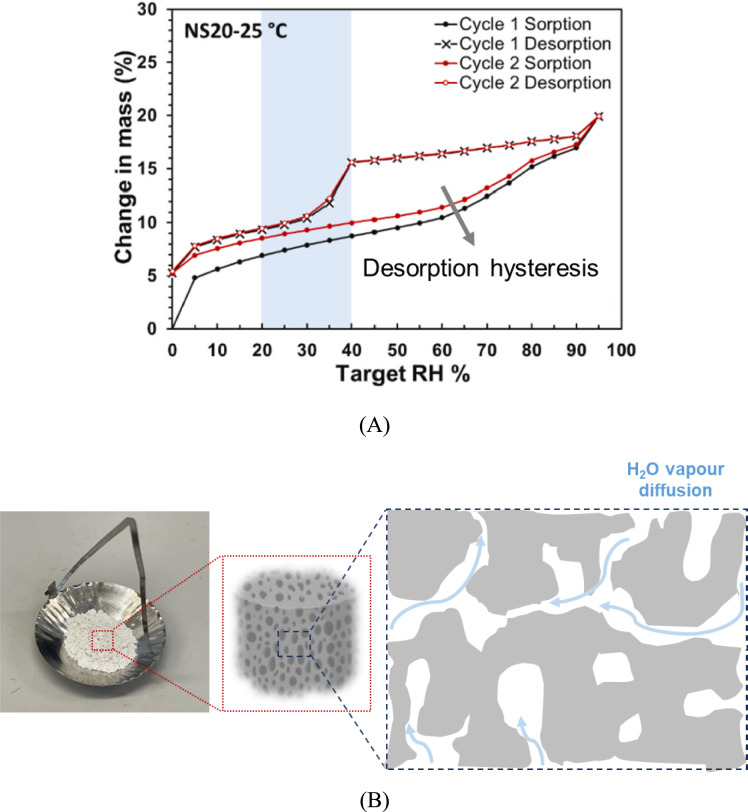
**A** A typical two-cycle water sorption–desorption isotherm of geopolymer-based thermochemical energy storage materials, **B** powdered geopolymer samples in DVS sample holder for characterisation

The vapour sorption kinetics of the material can be monitored with the dynamic vapor sorption (DVS) at given relative humidity and selected temperature. Since the method utilises water vapour to determine the sorption capacity of the material under certain conditions, it is not commonly used for studying the carbonation/decarbonation and hydroxylation/dehydroxylation processes. Ke and Baki [32] used DVS to study to study water sorption/desorption kinetics, such as water uptake capacity and diffusion coefficient, in addition to their cyclic sorption/desorption capacity (Fig. 5A). Additionally, the method of producing water vapour and recovering condensation energy significantly influences the maximum energy efficiency [3, 6].

#### Thermal stability and activation energy

Thermogravimetric analysis (TGA) is commonly used to study carbonation and decarbonation reactions, as it enables clear identification of mass changes associated with calcination and carbonation processes in the material [60]. For other cementitious materials, TGA is widely applied to determine water content [42], and in the case of ettringite, which is particularly susceptible to carbonation, it is also used to evaluate thermal stability [41]. To assess thermal energy storage density, differential scanning calorimetry (DSC) is typically used in combination with TGA, providing complementary information on heat flow and mass change during thermal cycling [32].

**Fig. 6:**
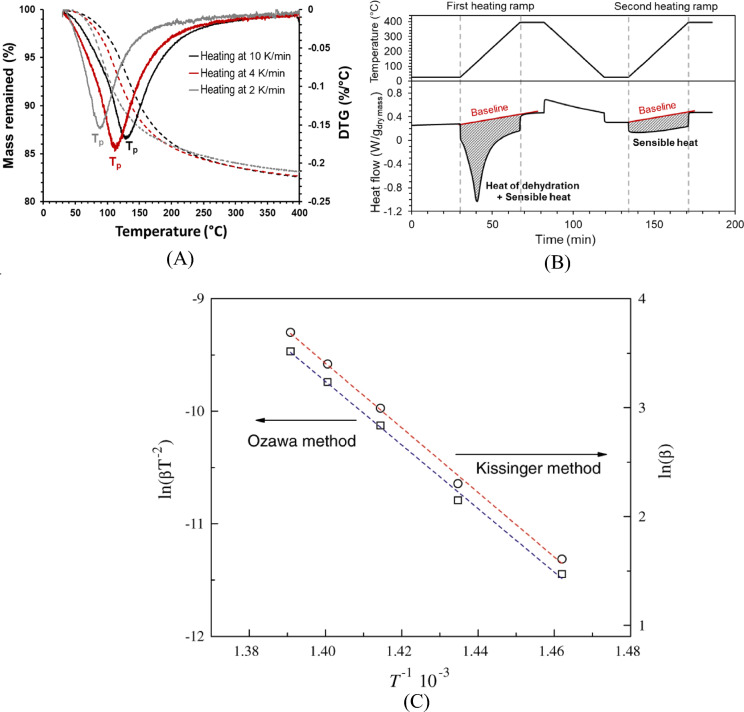
**A** Thermogravimetric results of a typical geopolymer N-A-S-H gel at three different heating rates, Tp refers to the peak decomposition temperature, from [32]. **B** DSC results a typical geopolymer N-A-S-H gel during two heating ramps as a function of time, from [32]. **C** An illustration of the linearisation curves of Ozawa method and Kissinger method used to determine the activation energy of dehydration of the assessed samples, from [72]

The activation energy of dehydrated thermochemical energy storage materials can be determined using both the Kissinger method [70] and the Ozawa method [71], as expressed by the Eqs. 9 and 10:

9 $$E_{{{\text{activation}}}} = R\frac{{d\left( {\ln \left( {\beta /T_{p}^{2} } \right)} \right)}}{{d\left( {1/T_{p} } \right)}} \left( {{\text{Kissinger}}\;{\text{ method}}} \right)$$

10 $$E_{{{\text{activation}}}} = - 0.4567 \times R\frac{{d\left( {\log \left( \beta \right)} \right)}}{{d\left( {1/T_{p} } \right)}} \left( {{\text{Ozawa }}\;{\text{method}}} \right)$$

where *β*, *T*_*p*_, and *R* are heating rate (K/min,) peak temperature (K) and gas constant (*R* = 8.314 J⋅K^−1^ mol^−1^). Figure 6A illustrates the differential thermogravimetric (DTG) results of geopolymer samples (primarily consist of N-A-S-H gels) under three different heating rates and the determined peak temperature (T_p_) values. Figure 6B demonstrate an example of a typical two-cycle DSC measurement, where both the sensible heat capacity (at dry state) and the heat of hydration can be determined [32]. The activation energy of dehydration then can be calculated using the linearisation curves of Kissinger method or the Ozawa method (Fig. 6C).

### Reactor level

#### Reactor systems

**Fig. 7:**
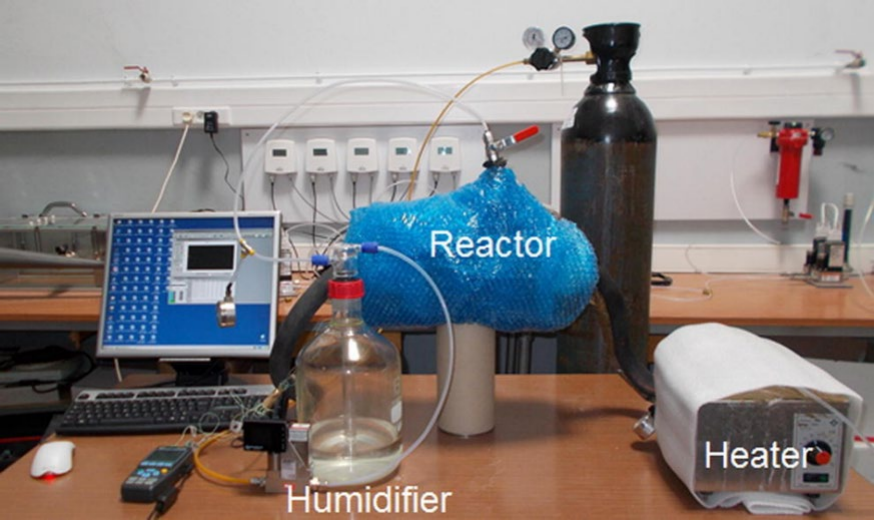
A lab scale reactor for initial determination of the optimal testing conditions [37]

**Fig. 8:**
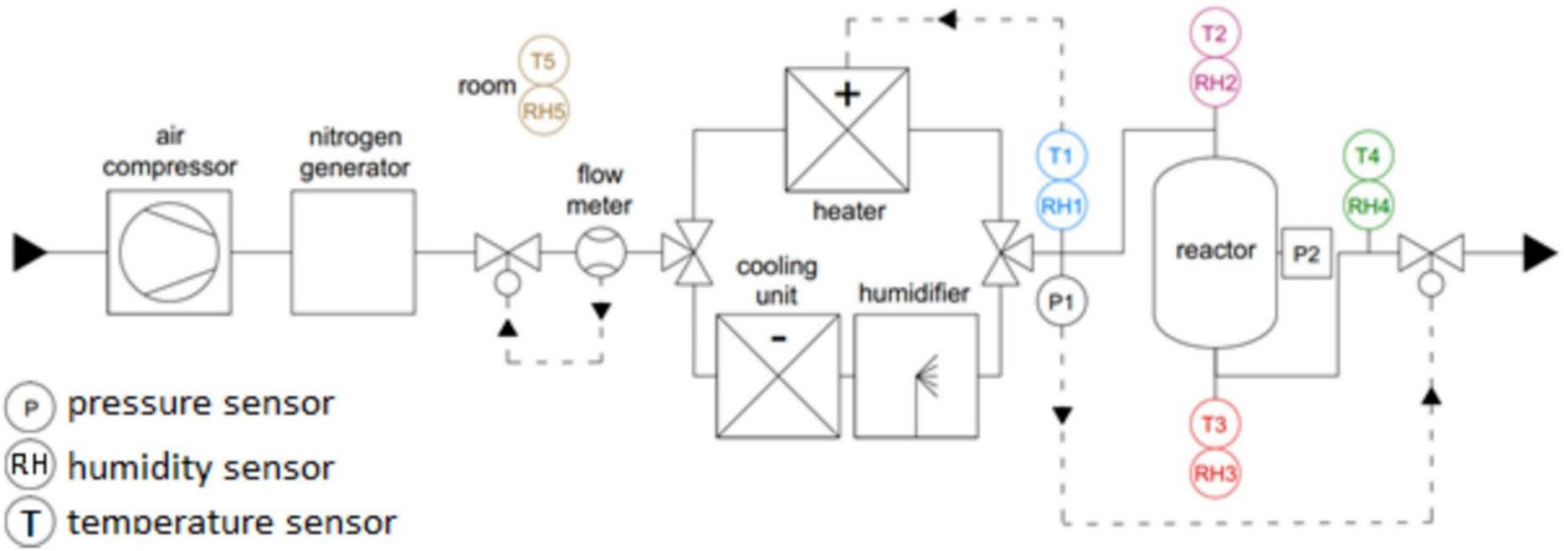
Schematic representation of a prototype TCES reactor system designed for ettringite-based thermochemical energy storage materials [51]

Chemical reactors are used to perform thermochemical energy storage. A recent review conducted by Solé et al. critically assessed the different types of chemical reactors for thermochemical energy storage [15], including packed bed reactors, fluidised bed reactors, open and closed reactors. The effective design of suitable reactors depending on the kinetic and thermochemical data of the chosen feedstock materials, as well as the working temperature range, associated cost, and durability of the materials. Two large groups of reactor systems can be distinguished, namely the open and closed reactors [3, 73]. In the first one, the reactant material is not isolated from the environment [74, 75]. Figures 7 and 8 illustrate the lab-scale thermochemical energy storage reactor [37] and a pilot-scale prototype reactor system [76].

The efficiency of the packed bed reactor strongly depends on the heat transfer rate (Schaube et al., 2011), which can be improved with higher effective thermal conductivity of the reactant particles [46]. Fluidized bed reactors promise much larger heat transfer coefficients. However, the fluidization of the material also requires large gas volume flows, which reduces the energy efficiency of the storage process [45]. Current research in reactor design for the calcium oxide/hydroxide system is mainly focused on moving and fluidised bed concepts, in preference to packed bed concepts, due to the necessity to reduce reactor cost [77].

#### Particle size of the reactant

Increasing the material-fluid exchange surface area in the thermochemical reactor can improve the efficiency of heat exchanges and the storage performance of the ettringite material [30]. Thus, the particle size of the bed material in the reactor plays a major role in the efficiency of the process by facilitating maximum gas diffusion [42]. Figure 6 shows the examples of zeolite and SrCl_2_-cement composite particles. The smaller the particle size, the higher sorption rate and hence the higher energy generation rate [42]. The same was also noted by N'Tsoukpoe, Restuccia [78], while [79] found that the wider particle size range significantly improved the thermal response of the materials, through improved packing. Similarly, the diffusion of CO_2_ molecules through the pores of the CaO particles has also been found to influence the efficiency of the carbonation reactor systems, as intraparticle pore diffusion hinders carbonation for particles larger than about 300 µm, with the ideal particle size to capture CO_2_ being 100–300 µm [60]. In other studies 50 μm was considered the threshold [59]. In [46] the average size of the Mg(OH)_2_ used was 240 μm, similarly to the 250 μm reported in [10]. On the other hand, pore-plugging is an important phenomenon that can limit gas–solid reactions, particularly if the pore size is not sufficiently large [60]. This is more pronounced when carbonation/decarbonation reactors are used, as carbonation conditions lead to a very fast buildup of a thick CaCO_3_ product layer on the surface of the CaO particles [60].

#### Gas flow rate

**Fig. 9:**
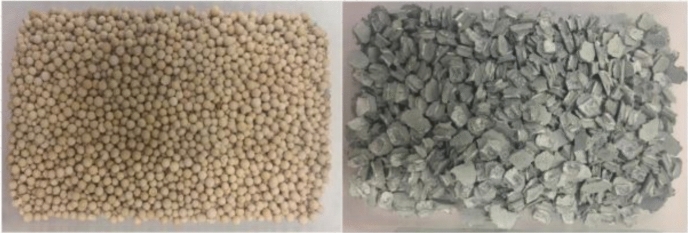
Zeolite (left) and SrCl_2_-cement composite (right) particles [42]

In the thermochemical energy storage reactor, the gas flow rate also plays a crucial role in determining the energy storage performance by controlling the water adsorption kinetics in thermochemical salts and composites [40, 41, 68] (Fig. 9). For cement-based composites with porous microstructures, their pore structures and pore tortuosity also play important roles. At high gas flow rates, the water molecules in the gas flow can reach the surface of thermochemical energy storage materials more quickly due to reduced external mass transfer resistance, generating a steeper surface concentration gradient. These phenomena can provide a stronger driving force for adsorption but may also reduce the overall degree of hydration if the humid gas flow is allowed to pass through the materials too quickly [25, 40, 68]. A slower flow rate of the humid gas can promote even water adsorption onto the thermochemical energy storage materials within the reactor but might result in lower water adsorption kinetics and therefore lower heat release rate and slower temperature rise. To achieve optimal thermochemical energy storage performance at the reactor level, optimising the gas flow rate through experimental trials is necessary.

## Recommendations and future research

To advance the field of thermochemical energy storage (TCES) using cementitious materials, several key areas require further research. First, multi-scale material characterization and examination are essential for designing and optimizing cement-based TCES materials. This involves not only understanding fundamental thermochemical properties at the material level, such as reaction kinetics, energy storage capacity, and cyclic stability, but also investigating the performance of these novel composite materials at the small reactor level. Comparing results from both material and small reactor levels will provide valuable insights into the practical energy storage performance and efficiency. Moreover, while significant progress has been made at the material and small reactor scales, future research should also focus on evaluating the performance of cementitious TCES materials under real-world, dynamic environmental conditions. This includes conducting system-level measurements and evaluations, such as the energy consumption of the operating system (i.e., electric heater, hot water pump, humidifier) [80], heat loss during operation, overall energy efficiency [81], and estimated cost of electricity [82], etc. Investigating the effects of fluctuating temperatures, humidity, and varying thermal loads is also crucial for translating laboratory successes into reliable, scalable, and resilient energy storage solutions for practical applications. Additionally, the optimisation of material synthesis and design processes will benefit from comprehensive analysis across all scales, including system-level evaluations.

Secondly, standardised testing programs and protocols are vital for ensuring consistency and comparability of results, which in turn supports the sustained development in this research area. This includes guidelines for sample preparation, testing conditions, and performance metrics, which can enable direct comparison between research carried out by different researchers. The standardised lab-scale reactor designs can also facilitate reproducibility and scalability of the TCES materials developed at the lab scale. The advancements in these areas can significantly advance the understanding and practical application of TCES using cementitious composites, contributing to more efficient and reliable energy storage systems.

Finally, in order to gain a full picture of the sustainability benefits of using cement-based materials for thermochemical energy storage, comprehensive life cycle assessment (LCA) studies of cementitious TCES materials should be conducted in future work. This includes comparing the sustainability of different TCES materials, such as ettringite, calcium aluminate cements, and geopolymers, taking into consideration their embodied carbon, operational lifespan, cyclability, and energy storage capacity. The comparative LCA studies can also support the selection of TCES material designs with the lowest life cycle environmental effects, enhancing energy resilience and sustainability in the built environment.

## Conclusions

This review highlights the significant progress and potential of cementitious materials, mainly ettringite, calcium aluminate cements, and geopolymers, for thermochemical energy storage (TCES) applications. These materials offer high volumetric energy density and minimal heat losses during charging and discharging cycles, making them ideal for long-term and seasonal energy storage. A multi-scale examination approach is crucial for advancing this field. At the material level, mineralogy characterisation and microstructure analysis are commonly used. The basic energy storage performance, the energy storage density, can be characterised by combining thermogravimetric analysis and differential scanning calorimetry. The dynamic water vapour sorption test can provide insights into the energy storage kinetics of the material, including the reaction kinetics and energy efficiency, which helps to close the gap between the material-level and reactor-level performance. However, in order to gain a better picture of the energy storage performance at the system-level, systematic experiments investigating the effect of reaction conditions (i.e., gas flow rate, inlet–outlet gas temperature and relative humidity) are also necessary. For future studies, standardization of testing techniques is necessary to ensure consistency and comparability across studies. Establishing standardized protocols for the characterising and testing of TCES materials will facilitate the advancement of this research area.

## References

[1] Alva G, Lin Y, Fang G (2018) An overview of thermal energy storage systems. Energy 144:341–378

[2] Salustro S et al (2024) Thermal characterization and cost analysis of cement-based composite materials for thermochemical energy storage. J Energy Storage 93:112308

[3] N’Tsoukpoe KE, Kuznik F (2021) A reality check on long-term thermochemical heat storage for household applications. Renew Sustain Energy Rev 139:110683

[4] Borri E, Zsembinszki G, Cabeza LF (2021) Recent developments of thermal energy storage applications in the built environment: a bibliometric analysis and systematic review. Appl Therm Eng 189:116666

[5] Gbenou TR, Fopah-Lele A, Wang K (2021) Recent status and prospects on thermochemical heat storage processes and applications. Entropy. 10.3390/e23080953 10.3390/e23080953 PMC8394121 34441093

[6] Kuznik F, Johannes K (2020) Thermodynamic efficiency of water vapor/solid chemical sorption heat storage for buildings: theoretical limits and integration considerations. Appl Sci. 10.3390/app10020489

[7] Metrane A et al (2022) Water vapor adsorption by porous materials: from chemistry to practical applications. J Chem Eng Data 67(7):1617–1653

[8] Ma Z, Bao H, Roskilly AP (2019) Seasonal solar thermal energy storage using thermochemical sorption in domestic dwellings in the UK. Energy 166:213–222

[9] Hongois S et al (2011) Development and characterisation of a new MgSO_4_−zeolite composite for long-term thermal energy storage. Solar Energy Mater Solar Cells 95(7):1831–1837

[10] Setoodeh Jahromy S et al (2019) Fly ash from municipal solid waste incineration as a potential thermochemical energy storage material. Energy Fuels 33(7):5810–5819

[11] Afflerbach S et al (2017) Semipermeable encapsulation of calcium hydroxide for thermochemical heat storage solutions. Sol Energy 148:1–11

[12] Lefebvre D, Tezel FH (2017) A review of energy storage technologies with a focus on adsorption thermal energy storage processes for heating applications. Renew Sustain Energy Rev 67:116–125

[13] O’Dwyer E et al (2019) Smart energy systems for sustainable smart cities: current developments, trends and future directions. Appl Energy 237:581–597

[14] Zhang Y, Wang R (2020) Sorption thermal energy storage: concept, process, applications and perspectives. Energy Storage Mater 27:352–369

[15] Solé A, Martorell I, Cabeza LF (2015) State of the art on gas–solid thermochemical energy storage systems and reactors for building applications. Renew Sustain Energy Rev 47:386–398

[16] Tatsidjodoung P, Le Pierrès N, Luo L (2013) A review of potential materials for thermal energy storage in building applications. Renew Sustain Energy Rev 18:327–349

[17] N’Tsoukpoe KE et al (2009) A review on long-term sorption solar energy storage. Renew Sustain Energy Rev 13(9):2385–2396

[18] Ding Y, Riffat SB (2013) Thermochemical energy storage technologies for building applications: a state-of-the-art review. Int J Low-Carbon Technol 8(2):106–116

[19] Liu Z et al (2013) An analytical model for determining the relative electrical resistivity of cement paste and C-S–H gel. Constr Build Mater 48:647–655

[20] Scapino L et al (2017) Sorption heat storage for long-term low-temperature applications: a review on the advancements at material and prototype scale. Appl Energy 190:920–948

[21] ElBahloul AA et al (2022) Recent advances in multistage sorption thermal energy storage systems. J Energy Storage 45:103683

[22] Khadim N, Stéphane G, Martin C (2018) Thermal energy storage based on cementitious materials: a review. AIMS Energy 6(1):97–120

[23] Lizana J et al (2017) Advances in thermal energy storage materials and their applications towards zero energy buildings: a critical review. Appl Energy 203:219–239

[24] Donkers PAJ et al (2017) A review of salt hydrates for seasonal heat storage in domestic applications. Appl Energy 199:45–68

[25] Clark R-J, Mehrabadi A, Farid M (2020) State of the art on salt hydrate thermochemical energy storage systems for use in building applications. J Energy Storage 27:101145

[26] Johannes K et al (2015) Design and characterisation of a high powered energy dense zeolite thermal energy storage system for buildings. Appl Energy 159:80–86

[27] Ortiz C et al (2018) Carbonation of limestone derived CaO for thermochemical energy storage: from kinetics to process integration in concentrating solar plants. ACS Sustain Chem Eng 6(5):6404–6417

[28] Wang K et al (2022) A review for Ca(OH)2/CaO thermochemical energy storage systems. J Energy Storage 50:104612

[29] Ndiaye K, Cyr M, Ginestet S (2017) Durability and stability of an ettringite-based material for thermal energy storage at low temperature. Cem Concr Res 99:106–115

[30] Ndiaye K, Ginestet S, Cyr M (2018) Experimental evaluation of two low temperature energy storage prototypes based on innovative cementitious material. Appl Energy 217:47–55

[31] Honorio T et al (2021) Ettringite hysteresis under sorption from molecular simulations. Cem Concr Res 150:106587

[32] Ke X, Baki VA (2021) Assessing the suitability of alkali-activated metakaolin geopolymer for thermochemical heat storage. Microporous Mesoporous Mater 325:111329

[33] Skevi L et al (2023) The effect of salt-impregnation on thermochemical properties of a metakaolin geopolymer composite for thermal energy storage. In: International RILEM conference on synergising expertise towards sustainability and robustness of cement-based materials and concrete structures. Springer Nature Switzerland, Cham

[34] Lavagna L et al (2020) Cementitious composite materials for thermal energy storage applications: a preliminary characterization and theoretical analysis. Sci Rep 10(1):12833 32733042 10.1038/s41598-020-69502-0 PMC7393370

[35] Teixeira P, Afonso E, Pinheiro CIC (2022) Tailoring waste-derived materials for Calcium-Looping application in thermochemical energy storage systems. J CO2 Util. 10.1016/j.jcou.2022.102180

[36] Struble LJ, Brown PW (1986) Heats of dehydration and specific heats of compounds found in concrete and their potential for thermal energy storage. Solar Energy Mater 14(1):1–12

[37] Ndiaye K, Ginestet S, Cyr M (2017) Modelling and experimental study of low temperature energy storage reactor using cementitious material. Appl Therm Eng 110:601–615

[38] Ndiaye K, Cyr M, Ginestet S (2020) Development of a cementitious material for thermal energy storage at low temperature. Constr Build Mater 242:118130

[39] Kaufmann J, Winnefeld F (2019) Seasonal heat storage in calcium sulfoaluminate based hardened cement pastes – experiences with different prototypes. J Energy Storage 25:100850

[40] Chen B et al (2021) Characterization of an ettringite-based thermochemical energy storage material in an open-mode reactor. J Energy Storage 33:102159

[41] Chen B et al (2021) Investigation on ettringite as a low-cost high-density thermochemical heat storage material: thermodynamics and kinetics. Solar Energy Mater Solar Cells 221:110877

[42] Clark R-J, Farid M (2021) Experimental investigation into the performance of novel SrCl_2_-based composite material for thermochemical energy storage. J Energy Storage 36:102390

[43] Clark R-J, Farid M (2021) Hydration reaction kinetics of SrCl2 and SrCl_2_-cement composite material for thermochemical energy storage. Solar Energy Mater Solar Cells 231:111311

[44] Funayama S et al (2019) Composite material for high-temperature thermochemical energy storage using calcium hydroxide and ceramic foam. Energy Storage 1(2):e53

[45] Schmidt M, Linder M (2020) A Novel Thermochemical Long Term Storage Concept: Balance of Renewable Electricity and Heat Demand in Buildings. Front Energy Res 8:137

[46] Yan J, Yang BW, Zhao CY (2022) Investigation of hydration/dehydration processes in a fluidized bed reactor using MgO/Mg(OH)_2_ thermochemical energy storage system. Sol Energy 231:630–645

[47] Humphries TD et al (2019) Dolomite: a low cost thermochemical energy storage material. J Mater Chem A 7(3):1206–1215

[48] Moreno V et al (2022) Albero: an alternative natural material for solar energy storage by the calcium-looping process. Chem Eng J 440:135707

[49] Chen B et al (2019) Physicochemical properties of ettringite/meta-ettringite for thermal energy storage: review. Solar Energy Mater Solar Cells 193:320–334

[50] Chen B et al (2020) Comparative kinetics study on carbonation of ettringite and meta-ettringite based materials. Cem Concr Res 137:106209

[51] Beaupere N et al (2024) Experimental study of a thermochemical energy storage system operating at low temperature with ettringite-based materials. Sol Energy 282:112927

[52] Richter M et al (2018) A systematic screening of salt hydrates as materials for a thermochemical heat transformer. Thermochim Acta 659:136–150

[53] Jeon IK et al (2023) Effects of shape-stabilized phase change materials in cementitious composites on thermal-mechanical properties and economic benefits. Appl Therm Eng 219:119444

[54] White CE et al (2010) The effects of temperature on the local structure of metakaolin-based geopolymer binder: a neutron pair distribution function investigation. J Am Ceram Soc 93(10):3486–3492

[55] Mahon D, Claudio G, Eames PC (2017) An experimental investigation to assess the potential of using MgSO4 impregnation and Mg2+ ion exchange to enhance the performance of 13X molecular sieves for interseasonal domestic thermochemical energy storage. Energy Convers Manage 150:870–877

[56] Shkatulov AI et al (2020) Stabilization of K2CO3 in vermiculite for thermochemical energy storage. Renew Energy 150:990–1000

[57] Permyakova A et al (2017) Design of salt–metal organic framework composites for seasonal heat storage applications. J Mater Chem A 5(25):12889–12898

[58] Yang Y et al (2022) Thermochemical heat storage and optical properties of red mud/Mn co-doped high alumina cement-stabilized carbide slag in CaO/CaCO_3_ cycles. Fuel Process Technol. 10.1016/j.fuproc.2022.107419

[59] Durán-Olivencia FJ, Espín MJ, Valverde JM (2020) Cross effect between temperature and consolidation on the flow behavior of granular materials in thermal energy storage systems. Powder Technol 363:135–145

[60] Benitez-Guerrero M et al (2017) Large-scale high-temperature solar energy storage using natural minerals. Solar Energy Mater Solar Cells 168:14–21

[61] Ortiz C et al (2021) Solar combined cycle with high-temperature thermochemical energy storage. Energy Convers Manag 241:114274

[62] Grasa G et al (2009) Application of the random pore model to the carbonation cyclic reaction. AIChE J 55(5):1246–1255

[63] Benitez-Guerrero M et al (2018) Calcium-looping performance of mechanically modified Al2O3-CaO composites for energy storage and CO2 capture. Chem Eng J 334:2343–2355

[64] Arcenegui-Troya J et al (2021) Kinetics and cyclability of limestone (CaCO3) in presence of steam during calcination in the CaL scheme for thermochemical energy storage. Chem Eng J 417:129194

[65] Teng L et al (2020) Modified Ca-looping materials for directly capturing solar energy and high-temperature storage. Energy Storage Mater 25:836–845

[66] Cosquillo Mejia A et al (2020) Experimental analysis of encapsulated CaO/Ca(OH)2 granules as thermochemical storage in a novel moving bed reactor. Appl Therm Eng 169:114961

[67] Ogorodova LP et al (2021) Physicochemical and thermochemical study of Ettringite. Geochem Int 59(12):1188–1197

[68] Reynolds J et al (2024) Optimisation of CaCl2 impregnated expanded graphite and alginate matrices – targeted salt loading. Energy Convers Manage 302:118145

[69] Bird JE et al (2020) Thermal properties of thermochemical heat storage materials. Phys Chem Chem Phys 22(8):4617–4625 32051979 10.1039/c9cp05940g

[70] Kissinger HE (1956) Variation of peak temperature with heating rate in differential thermal analysis. J Res Natl Bur Stand 57(4):2712

[71] Ozawa T (1970) Kinetic analysis of derivative curves in thermal analysis. J Therm Anal 2(3):301–324

[72] Yu W et al (2010) Highly efficient method for preparing homogeneous and stable colloids containing graphene oxide. Nanoscale Res Lett 6(1):47 27502669 10.1007/s11671-010-9779-7 PMC3211982

[73] Abedin AH, Rosen MA (2012) Closed and open thermochemical energy storage: energy- and exergy-based comparisons. Energy 41(1):83–92

[74] Jänchen J et al (2015) Performance of an open thermal adsorption storage system with Linde type A zeolites: beads versus honeycombs. Microporous Mesoporous Mater 207:179–184

[75] Zettl B, Englmair G, Steinmaurer G (2014) Development of a revolving drum reactor for open-sorption heat storage processes. Appl Therm Eng 70(1):42–49

[76] Zondag H et al (2013) Prototype thermochemical heat storage with open reactor system. Appl Energy 109:360–365

[77] Gollsch M et al (2020) Investigation of calcium hydroxide powder for thermochemical storage modified with nanostructured flow agents. Sol Energy 201:810–818

[78] N’Tsoukpoe KE et al (2014) The size of sorbents in low pressure sorption or thermochemical energy storage processes. Energy 77:983–998

[79] Walsh S et al (2020) Assessing the dynamic performance of thermochemical storage materials. Energies. 10.3390/en13092202

[80] Yang H et al (2024) Experimental investigation of a thermochemical energy storage system based on MgSO4-silica gel for building heating: adsorption/desorption performance testing and system optimization. Energy Convers Manage 301:118000

[81] Airò Farulla G et al (2020) A review of thermochemical energy storage systems for power grid support. Appl Sci 10(9):3142

[82] Bajaj I, Peng X, Maravelias CT (2024) Screening and property targeting of thermochemical energy storage materials in concentrated solar power using thermodynamics-based insights and mathematical optimization. RSC Sustain 2(4):943–960

